# Analytical Methods Used for the Detection and Quantification of Benzodiazepines

**DOI:** 10.1155/2019/2035492

**Published:** 2019-09-05

**Authors:** Zidane Qriouet, Zineb Qmichou, Nadia Bouchoutrouch, Hassan Mahi, Yahia Cherrah, Hassan Sefrioui

**Affiliations:** ^1^Medical Biotechnology Center, Moroccan Foundation for Science, Innovation & Research (MAScIR), Rabat, Morocco; ^2^Laboratoire de Pharmacologie et Toxicologie, Faculté de Médecine et de Pharmacie, Université Mohammed V-Souissi, Rabat, Morocco

## Abstract

The prescription of psychotropic drugs, especially benzodiazepines (BZDs), occupies a preponderant place in the management of mental illnesses. Indeed, the BZDs have been used in different therapeutic areas including insomnia, anxiety, seizure disorders, or general anesthesia. Unfortunately, these drugs are present in the illegal street market, leading to a lot of drug abuse amongst some addicted users, road insecurity, and suicide. Hence, it has become essential to analyze the BZDs drugs in human biological specimens for drug abuse in forensic sciences. The present review provides a summary of sample preparation techniques (solid-phase extraction and Liquid-liquid phase extraction) and the methods for the detection and quantification of BZDs molecules in the commonly used biological specimens over the ten last years which may potentially lead to better and accurate evaluation of the physiological state of a given person. The commonly used methods for the detection and quantification of BZDs include nuclear magnetic resonance (NMR), chromatography (GC-MS, HPLC, and TLC), immunoassay (ELISA, RIA, LFA, CEDEA, FPIA, and KIMS), and electroanalytical methods (voltammetry and potentiometry).

## 1. Introduction

Benzodiazepines (BZDs) are a class of psychoactive drugs, introduced in the 1960s, creating a revolution in the field of anxiolytic drugs [1]. The list of the most prescribed BZDs in the world is categorized, according to their main property, as anticonvulsant, sedative, anxiolytic, amnesic, and hypnotic [2, 3]. BZDs were amongst the most prescribed psychotropic drugs in western countries, particularly in France [4]. Their annual prevalence use is around 2% to 17% [4, 5] and varies with countries and from one scientific study to another. The abuse or misuse of BZDs is one of the potential serious social problems worldwide. Their prescription must be reassessed after a short period (12 weeks), because long-term BZDs use has also been described as causing cognitive effects (increasing incidence of dementia), dependence, and withdrawal [6–8]. Indeed, the official international recommendations concerning the use of this therapeutic BZDs molecules are frequently updated, emphasizing short and uninterrupted prescription periods in order to avoid possible abuse of these drugs.

To prevent the BZDs increasing incidence of abuse in the world, the researchers focus on the development of innovative, highly sensitive, and accurate methods to analyze the BZDs and their metabolites. Indeed, the determination of BZDs in biological fluids is essential in clinical assays as well as in forensics and toxicological studies.

The commonly used biological specimens for the analysis of BZDs are blood, urine, and saliva. The major factors evaluated during this analysis are related to the presence or absence of the target BZDs molecules or their related metabolite in the tested samples.

Currently, there are 2 types of BZDs analysis: 1, the screening (qualitative or semiquantitative methods) and 2, the dosage (quantitative methods). However, a toxicological screening usually involves several screening and dosing methods.

Many techniques more and more efficient do exist (GC-MS, HPLC, RMN, etc.). The possibilities of detection and quantification of BZDs will still depend on the methods available in each laboratory. Indeed, the clinician or the scientist must know which ones are accessible to him, their limits of sensitivity and accuracy, and their time constraints in order not to prescribe them unnecessarily. Thus, the method allowing quick, sensitive, and cost-effective detection of BZDs does not exist yet.

Our review covers the analytical methods for BZDs determination and sample preparation techniques used in the studies published over the past ten years.

## 2. Biological Specimens Commonly Used for the Analysis of BZDs

The detection and quantification of BZDs can be done in different biological matrices including human breast milk [9], saliva [10–12], blood [13–15], blood serum [16], plasma [9], urine [14, 17–20], nails [21], hair [22], meconium [23], edible animal tissues and feed [24], and expired air [25]. However, it is important to keep in mind that whatever the biological matrix used for the screening, the BZDs concentrations depend on many factors, namely, the consumed dose, the quality of the product, the mode of consumption, the metabolism of the consumer, the body weight of the user, and his state of health.

In the present review, we will discuss the most commonly used biological matrices for psychotropic drug analysis [26]. A special focus will be on saliva, urine, and blood matrices.

### 2.1. Saliva

Saliva is one of the interesting biological specimens for detecting a recent psychotropic drugs intake compared to urine [27]. It is considered as one of the major arguments in favor of its use in health, at work or at the roadside by the police in the detection of narcotics used by drivers of vehicles involved in traffic accidents [28, 29]. Indeed, saliva was approved as the screening method [30] in 2011 by the Substance Abuse and Mental Health Services Administration (SAMHSA, USA).

Amongst the drugs detected in human saliva, we have the amphetamines, 3,4-methylenedioxy-methamphetamine (MDMA), cocaine, opiates (heroin and codeine), cannabis, and BZDs [10–12, 19, 28, 31–34].

The main benefits of using saliva samples for psychotropic drug screening include its noninvasive nature, ease of sampling, handling does not raise difficulties with the intimacy of the person, and adulteration is very difficult. In fact, only a visual checkup by trained staff is required [35]. In addition, the salivary screening assay has the advantage of testing for parent molecules rather than metabolites. The pharmacokinetic profile of the molecules appears to be parallel in saliva and plasma, although the ratio of blood and salivary levels is inconsistent for a number of molecules. Hence, these pharmacokinetic characteristics represent a significant advantage when one seeks to establish a formal link between an exposure and the occurrence of an accident. It is not necessarily the same when one seeks to establish the existence of an impregnation and a longer detection time is more informative [35].

However, the saliva collecting method is very crucial and can influence the results. The contamination of the saliva sample by other substances such as certain foods, condiments, drinks, or bacteria cannot be formally excluded [36]. Also, there is no standard method for treating and collecting samples before analysis, and the choice of sampling device and the treatment of the saliva have been approached very differently by different authors [11, 12, 37].

### 2.2. Urine

Urine drug tests are the most common types of tests used among medical professionals. Many countries adopted these tests for road control, and many jobs now require a screening in order to apply. In research area, many studies used urine as biological matrix for drug analysis [14, 17–20, 38]. Indeed, the search for illicit drugs in the urine provides information on chronic or recent consumption. Yet, urine-screening tests are more difficult to implement, because the sampling presents major constraints. Urine tests are more common because they are noninvasive, are fast, offer the advantage of providing a large sample volume, and are able to qualitatively detect a wide range of substances including BZD. These tests offer also a lengthy amount of detection time; thus, the detection of drugs depends on the frequency and quantity of drugs used (1 to 4 days for most drugs), the development and validation of more sensitive and accurate analysis methods, the cutoff levels, the standard recognized protocols, and the standards of practice.

However, the urine needs to be properly stored to provide stable and valid results, and due to the privacy of providing samples, it can be altered before the analysis. To respect the person's privacy and also to avoid adulteration of the levy, it is necessary to impose adapted premises, trained personnel, examination of the color, density, measurement of the pH, and temperature of the sample immediately after it was carried out.

### 2.3. Blood

Blood is probably the only medium with the potential to indicate whether an individual is under the influence of BZD, or not, at the time of collection. It is considered as an essential element in the control of drug abuse in the workplace.

From a practical point of view, blood sample is the most restrictive to collect, compared to saliva or even urine. Indeed, it needs to be performed by qualified medical personnel in a laboratory. However, this process takes time, and sometimes, it can mean the difference in accuracy between a positive or negative test.

Blood tests can be performed to quantify the levels of certain BZDs and their metabolites but are more rarely practiced because of their invasive procedure.

Blood unlike urine has the advantage of being impossible to be impaired, and moreover, there is a proven relationship between the amount absorbed and the blood level and therefore effects on the central nervous system (dose/concentration relationship and dose/effect) [39]. The detection window in the blood is narrower than urine, and the concentrations are lower. Therefore, a sensitive and a very specific confirmation technique is mandatory for the detection of BZDs and their metabolites in the blood like HPLC or LC-MS/MS [11, 17–19], gas chromatography, or GC-Mass spectrometry [10, 13–15, 18].

These matrices contain a multitude of substances endogenous (proteins in the blood or fatty acids in urine) in amounts well above those compounds and their metabolites to be quantified. Many endogenous compounds have reactive functional groups (such as the carboxylic functions of amino acids or fatty acids) that can participate in derivatization reactions and interfere with the analysis of the compounds of interest. Preprocessing of samples is therefore a fundamental step for this type of analysis. In this regard, modern isolation techniques, e.g., solid-phase extraction, are necessary [13].

## 3. Sample Preparation (Pretreatment and Extraction)

Pretreatment of the sample aims to isolate and concentrate the xenobiotic (*s*) in a matrix while extracting as little as possible of the endogenous compounds prior to analysis. This is most certainly the most important step in the analytic process. The diversity of biological samples, entrusted to toxicological laboratories for analysis, show complex matrices (blood, urine, hair, saliva, meconium, etc.). From this observation, it is well known why good preparation has a direct influence on the limit of detection, repeatability, and reproducibility of the analysis. Over the years, various procedures have been developed including liquid-liquid extraction (LLE), solid-phase extraction (SPE), molecularly imprinted solid-phase extraction (MISPE), microextraction (SPME and LPME), supercritical fluid extraction (SFE), online solid-phase extraction, ASPEC (automated sample preparation with extraction columns) system, column switching, dispersive liquid-liquid microextraction (DLLME), nonextraction procedures (dialysis of biofluids using a semipermeable gold membrane direct injection of crude samples after protein precipitation), and fabric phase sorptive extraction (FPSE) that combines the extraction of SPME/SPE mode into a single technology platform. In this review, we will describe the LLE and the SPE methods.

### 3.1. Solid-Phase Extraction (SPE)

Solid-phase extraction is based on the sharing of compounds between a liquid phase, the sample, and a stationary phase, the adsorbent. It usually consists of four stages.

The first step is the conditioning of the stationary phase. It allows it to be wetted by means of an organic solvent and to activate the sites of retention, the seat of the molecular interactions. A hydrophobic support is conditioned by an organic solvent (most often methanol) and then by a solvent whose ionic and pH characteristics are as close as possible to the solvent of the sample (generally water). The second step is the deposit of the sample. The goal is to cause a quantitative retention of analytes of interest on the stationary phase, while the maximum of interference is eliminated by simple nonretention. For maximum efficiency, the flow velocity of the sample should be moderate.

The next step is washing. It is not systematic; it aims to eliminate interferences weakly retained. It is necessary to choose solvents of weak eluent forces (e.g., solution methanol/water) to elute only the interferents. This step for so-called mixed phases can be multiplied by acting alternatively on one of the mechanisms, for example, first washing with a weak eluent strength solution for our analytes and then a second washing by modifying the pH of the mobile phase. These multiple washes improve very clearly the cleanliness of the extract contributing to the quality of the analysis. It is recommended at the end of this step to dry the support to evaporate traces of washing solvent. This step improves the extraction yield.

The last step is elution. It is preferable to use the solvent with the lowest possible eluting force capable of driving all the molecules of interest, thus avoiding eluting strongly retained interferents. The choice of the solvent is also guided by its ease of evaporation or its compatibility with the following analytical technique. It must nevertheless be as effective as possible; its volume must be low so as to obtain a very important preconcentration factor. The flow rate of the solvent must be slow to promote elution.

Finally, SPE has taken an important place in the preparation of samples over the years [13, 40]. The range of stationary phases and their packaging are regularly enriched. This extraction method makes it possible to easily extract compounds that are difficult to extract, because they are very polar, with organic solvents and that could therefore only be analyzed after a simple precipitation. In addition, its automation that exists in different forms promises its wide utilization in the future by many laboratories. [13, 40, 41].

### 3.2. Liquid-Liquid Extraction (LLE)

Liquid-liquid extraction (LLE) methods allow the transfer of a solute initially contained in a liquid phase to another immiscible liquid phase. They are commonly used in pharmacology/toxicology to purify and concentrate samples prior to chromatographic or other analyses [20, 42]. Various physicochemical parameters govern the production of an LLE, specific to the solvents used and to the solutes to be extracted. The knowledge of certain properties of the solvent such as its miscibility with water, acidity constant, dielectric constant, dipole moment, density, volatility, and its toxicity will allow the choice of this solvent alone or in mixture for the extraction of a given substance. In the same way, the knowledge of the properties of the solute such as the structure, the acidity constant, the lipophilic, the nature, and the complexity of the matrix in which it is will make it possible to optimize the extraction, whose efficiency will be evaluated by the extraction yield. The mastery of all these variables will allow the operator to optimize the LLE steps when developing analysis methods in pharmacology/toxicology.

## 4. Methods Commonly Used for the Analysis of BZDs in Biological Specimens

Many methods for the determination of BZDs in biological samples have been reported in the literature. Some are qualitative and some others are quantitative methods. Indeed, developed methods are classified as chromatography (HPLC, TLC, and GC) [11–15, 18], immunoassays [38, 43–48], photometric (nuclear magnetic resonance and ultraviolet-visible) [20, 42], and electroanalytical methods (potentiometric, polarographic, and voltammetry) [49–51] have been increasingly used for drugs of abuse in forensic science.

Despite the fact that chromatographic methods still play a main role [52] in the detection and determination of BZDs in biological matrices [11, 12, 14, 15, 18], alternative methods like immunoassays [38, 53, 54] have been increasingly used for drugs of abuse in forensic science.

In case of suspected misuse or acute intoxication by BZDs, the toxicological analysis is often very useful for confirmation. The latter consists of three steps:

 
*Step 1*. Screening using immunoenzymological methods, which allow a quick identification of the class of the offending drug
 
*Step 2*. Identification by spectroscopic and/or chromatographic techniques, well adapted to the emergency but having certain limits
 
*Step 3*. Quantification of the BZDs molecule by chromatographic or spectroscopic adapted techniques

To get accurate and reliable results, relatively clean samples need to be analyzed. As a result, the pretreatment of the biological sample is an essential part of any analytical method. It allows improving the reproducibility of the analysis, lowering the limit of quantification of the method by decreasing background noise and concentration steps, improving the fidelity and accuracy of the analysis, and finally increasing the selectivity.

In this regard, modern isolation techniques, e.g., solid-phase microextraction (SPME) or LLE [20] which help to concentrate volatile or nonvolatile compounds in samples before GC or HPLC analysis [13, 20], solvent extraction after derivation, and stationary phases of grafted silica polar, are very important.

### 4.1. Screening/Detection Methods

The confirmation of BZDs consumption theoretically includes a screening test, followed, in case of positivity, by a confirmation test. Screening is usually done on company premises (workplaces) by “onsite” testing or in the laboratory by automated techniques. Overall, it is an immunological technique based on the recognition by a specific antibody (Ab) of the target BZD molecule (antigen) to be investigated. In these cases, biological media dedicated to screening are urine and saliva, although matrices such as blood or hair can be used.

There are currently many immunoenzymatic methods suitable for screening BZDs and their metabolites in body fluids. The principle lies in a competition between a labeled antigen and unlabeled antigen (BZD) against a specific antibody, and the major immunoassays are described below.

#### 4.1.1. Lateral Flow Immunoassay (LFIA)

The lateral flow immunoassay is a rapid and a simple paper-based devices used for the detection of a target molecule in liquid sample without the need for costly equipment. This method offers an interesting detection level for BZDs screening with low circulating concentrations. For example, in 2014, Toubou et al. [45] used the Oratect® III commercialized LFIA to detect alprazolam, prazepam, diazepam, and estazolam in whole blood with a detection limit of 60, 75, 25, and 15 ng/mL, respectively, and Berck et al. [46] used an inhouse LFIA and found that the observed positive cutoff for oxazepam was 400 ng/ml. Over the past decade, there has been a growing interest in the use of these innovative devices and saliva testing, particularly in the area of road safety [10, 43, 44]. Indeed, there are indications that reliability of these tests is improving and number of countries using them for roadside checks is increasing. However, their use in occupational health remains very limited at the moment.

In rapid detection tests, there is no perfect correlation between the results of drug tests in saliva, blood, or urine analysis. This may due to the difference in time between the moment of drug consumption and the testing time. For example, in 2013, Mohamed et al. [47] found that tobacco interferes with laminar flow immunoassays (LFIA) of urinary drug detection; therefore, all subjects must be questioned about their smoking status to avoid false positive results. Oral fluid cannot be seen as a substitute for blood or urine drug testing. Each specimen has its own distinct advantages and disadvantages. For example, if a driver consumed the medication just before the test, he or she could display a positive result on a blood or a saliva test, but not on a urine test. On the other hand, if he or she is a drug abuser before the test, he or she could show a positive result only to a urine test. Thus, drug-screening challenge arises not in the screening devices but rather in determining the best ways to proceed with impairments. Most of commercialized drug detection rapid tests use a LFIA based on four variables:

1. The threshold, the level of concentration from which the device is able to detect a BZD.
2. The sensitivity of the device or the test refers to the probability of obtaining a positive result for the presence of drugs in the body at the time of analysis. Indeed, the higher the sensitivity of the device, the lower the false negative rate.
3. The specificity, the measure of the probability of getting a negative result, but no drugs in the body of the driver at the time of the test. The higher the specificity of the device, the lower the false positive rate. A screening device designed for use in workplaces with high levels of sensitivity and specificity makes it possible to quickly identify people who have used drugs and to minimize the detention time of people without drugs in their homes or organization.
4. The last variable is related to the biological detection medium.

#### 4.1.2. Radioimmunoassay (RIA)

The radioimmunoassay (RIA) technique, as the name implies, achieves sensitivity through the use of radionuclides and specificity that is uniquely associated with immunochemical reactions. RIA is based on the competition between two antigens, which can bind to the same antibody. The radiolabeled, added in excess, and unlabeled antigens (tested molecule) compete for the limited binding sites on the antibody. The more the sample antigen is present, the less the radiolabeled antigen is able to bind to the antibody. The radiolabeled antigen must generally be present in low concentrations, because the quantity of molecule to be measured is usually small. Since the complex Ag-Ab is heavier than the one containing the unbound antigen, a centrifugation of the mixture will allow the separation into “free” and “bound” fractions and their radioactive counts measured. The concentration of test antigen can be calculated from the ratio of the bound and total antigen labels using a standard dose response curve. By measuring the radioactivity of the pellet, it is possible to determine the amount of radiolabeled Ag that has bound to Ab and therefore the concentration of Ag in the sample. Several authors have used this technique for the detection and quantification of benzodiazepines [55]. The most used isotopes in RIA are ^3^H, ^14^C, ^32^P, ^125^I, and ^57^Co. However, because of the long half-life of the first three and because their disintegration passes by the emission of beta particles, only ^125^I and ^57^Co are still used, with a clear preference for ^125^I, whose half-life is 60 days and emits easily detectable gamma particles. RIA technique is known for a low level of detection up to very low concentrations and high specificity. Although highly suitable for large series, the use of RIA is very rare in clinical laboratories, especially with the presence of enzyme-linked immunosorbent assay (ELISA) [56] and mainly because of the disadvantages inherent to the handling of radioisotopes. The majority of RIA assay formats recommend sample cleaning and concentration (particularly when analyte's concentration and assay sensitivity are low) [55].

#### 4.1.3. Competitive Enzyme-Linked Immunosorbent Assay (ELISA)

The enzyme-linked immunosorbent assay (ELISA) technique is an immunoenzymatic detection and quantification technique that makes possible to visualize an antigen-antibody reaction by means of a color reaction produced by the action on a substrate of an enzyme previously fixed to the antibody. ELISA has been used in many studies to detect BZDs in different biological specimens [24, 48, 57–59]. There are several different ELISA methods, namely, the competitive and the noncompetitive ELISA. Usually, BZDs screening is done by the competitive ELISA assays that is frequently used for the detection of small antigens containing a single epitope. The competition occurs between labeled (in known quantity) and unlabeled antigen of interest (BZDs) for a limited number of antibody sites. The signal generated by this assay will be inversely proportional to the concentration of unlabeled antigen in the sample which could be quantified using the standard curve which is prepared by performing a dilution series of a known concentration of the analyte across a range of concentrations near the expected unknown concentration. There are systems for increasing the sensitivity, which means to reduce the detection threshold of the constituents by using substrates giving a larger signal for the same amount of enzyme, for example, using conjugates with *β*-galactosidase revealed with a fluorogenic substrate or peroxidase conjugates with emission of a flash of light revealed by chemiluminescence.

The second approach is to amplify the signal by increasing the amount of enzyme in the Ag-Ab-enzyme complex. But since it is impossible to prepare more marked conjugates (with more enzyme molecules) at the risk of denaturing the activity of the Ab or enzymes, it is resorted to couplings allowing real scaffolding, as the avidin-biotin. Each stage of the scaffold amplifies the preceding one.

This test offers an interesting detection of BZDs with low concentrations; for example, in 2015, O'Connor et al. [48] have used Immunalysis® Benzodiazepine ELISA kit to test the crossreactivity with 10 BZDs including etizolam, nitrazepam, 7-aminoflunitrazepam, oxazepam, temazepam, chlorodiazepoxide, diazepam, phenazepam, desmethyldiazepam, and lorazepam in a sample blood. The pyrazolam and etizolam had a limit of detection of 0.0025 mg/L and a limit of quantitation of 0.005 mg/L.

#### 4.1.4. Cloned-Enzyme Donor Immunoassay (CEDIA)

Cloned-enzyme donor immunoassay for BZDs analysis is a single homogeneous phase immunoenzymatic method that uses recombinant DNA technology. This test uses the bacterial enzyme *β*-galactosidase previously split into two inactive fragments by genetic engineering. These fragments spontaneously reassociate to form a fully active enzyme that, upon reaction, fragments a substrate, producing a color change that can be measured by spectrophotometry. The drug (BZD) in the sample competes with the drug conjugated to one of the inactive fragments of *β*-galactosidase to bind to the Ab binding sites. If the drug is present in the sample, it attaches to the Ab, leaving the inactive fragments of the enzyme to form an active enzyme. If the sample does not contain a drug, the Ab binds to the conjugated drug of the inactive segment, hindering the reassociation of the inactive *β*-galactosidase fragments, which prevents the formation of an active enzyme. The amount of active enzyme formed and the resulting extinction variation are proportional to the amount of drug present in the sample. In order to improve the sensitivity of the test, an optional enzyme is added to hydrolyze the glycoconjugate metabolites of BZDs, thus favoring the detection of samples containing BZDs metabolites [38, 53, 54, 60–64].

Every laboratory has to validate the CEDIA test to use depending on the requirements individually and define cutoff values, for example, Musshoff et al. in 2013 [54] and Darragh et al. in 2014 [38] after they have optimized the set of samples (urine), the cutoff of the assays was 25 and 200 ng/ml, respectively.

#### 4.1.5. Fluorescence Polarization Immunoassay (FPIA)

The fluorescence polarization immunoassay uses the fluorescence polarization measurement emitted after excitation of a fluorescent substance by an equally polarized light beam. The degree of polarization of the emitted fluorescence directly depends on the amount of labeled ligand attached to the Ab. The sensitivity of FPIA technology is comparable to that of enzyme multiplied immunoassay technique (EMIT) methods. However, it has the main drawback of an unsuitable detection threshold for the lowest dose of BZDs especially triazolam and flunitrazepam, which are generally the most toxic ones. In fact, the results are positive only with toxic concentrations of BZDs, because a positive result is obtained for a concentration greater than 100 ng/ml, while the toxic concentrations of BZDs varied between 50 and 100 ng/ml [53]. From this point of view, the FPIA method has been less used in recent works.

#### 4.1.6. Kinetic Interaction of Microparticles in Solution (KIMS)

The kinetic interaction of microparticles in solution (KIMS) test is based on the kinetic interaction of microparticles in a given solution. For example, Abs recognizing BZDs are covalently bound to microparticles, and the drug derivative is linked to a macromolecule. The kinetic interaction of the microparticles in the solution is induced by the binding of the drug conjugate to the Ab on the microparticles and inhibited by the presence of BZDs in the sample. The drug conjugate and the BZDs in the sample compete for the binding sites of the BZDs Abs to the microparticles. The resulting kinetic interaction of microparticles is indirectly proportional to the amount of drug present in the sample [38].

The KIMS assay was performed by Darragh et al. [38] according to manufacturer instructions and optimized to a cutoff of 100 ng/mL, a sensitivity of 47%, and a specificity of 100%, although Bertol et al. in 2013 [53] validated the KIMS assay to a cutoff of 200 ng/mL, a sensitivity of 100%, and a specificity of 40%. Generally, every laboratory optimizes its test to use according to the personal needs.

### 4.2. Identification and Quantification Methods

Although screening tests are very important for BZDs analysis, they offer only a provisional result. Using any immunological technique, the risk of false positive by cross reaction with another drug is important, and therefore, it requires confirmation of positive samples by a quantitative method such as chromatographic or spectroscopic allowing unambiguous identification and exact determination of the concentrations of the BZD molecule. The biological specimen dedicated for BZDs confirmation remains the blood, but recent work describes potential effective confirmation techniques using saliva or urine [11, 12, 14, 17–19].

#### 4.2.1. Gas Chromatography Coupled to Mass Spectrometry (GC-MS)

The gas chromatography coupled to the mass spectrometry (GC-MS) method is generally considered among the reference methods for BZDs detection and quantification. This technique is the most powerful tool for identifying such drugs in biological media because of its high sensitivity and specificity. The actual analysis by GC-MS is preceded by a step of sample preparation. This step is long and difficult to be automatable. Hence, the identification is based on the specific detection of high mass ions of the substance to be analyzed. The search for the identity of the specific ions obtained is performed by comparison with a given reference library [10, 13, 15, 18, 60].

GC-MS offers the double advantage of quantification and formal identification of BZDs with often low detection limits (Table 1). In addition, the use of GC poses a number of problems mainly related to the thermolability of most BZDs that degrade rapidly in the absence of prior derivatization. However, this stage, often long and delicate, is only poorly suited to emergency toxicological analysis. Despite these difficulties, the GC-MS remains an interesting method when it comes to confirming an ambiguous diagnosis.

#### 4.2.2. High-Performance Liquid Chromatography (HPLC)

HPLC is the technique of choice for BZDs analysis and quantification [12, 65]. Compared with the GC method, HPLC does not expose molecules to thermal degradation and thus makes it possible to overcome derivatization reactions. A simple sample preparation step (liquid-liquid or solid-phase extraction) is usually sufficient. While detection by UV spectrometry is the most commonly used, coupling with mass spectrometry (HPLC-MS or MS-MS) is the method of choice for the identification and quantification of BZDs [11, 12, 60]. This method offers a particularly interesting detection level for the characterization and the determination of BZDs with very low circulating concentrations (Table 2). In addition, the automation of extraction procedures, particularly in the solid phase, generally allows the analysis in a very short time, compatible with the emergency.

#### 4.2.3. Thin-Layer Chromatography (TLC)

The thin-layer chromatography (TLC) is the simplest of the chromatographic methods. Indeed, it consists in placing on a sheet (paper, silica, or others) a stain and let it elute by soaking in a recommended solvent or a mixture of solvents (called eluent). The eluent diffuses along the support. After that, the stain migrates on the leaf, more or less quickly depending on the nature of the interactions, it undergoes on the part of the support and the eluent. The revelation is then done by colored reactions. The TLC method is much less used in emergency cases in toxicology. This technique can be quite fast (about half an hour) but lacks specificity and sensitivity and especially the interpretation is delicate [66, 67].

#### 4.2.4. Ultraviolet-Visible Spectroscopy

The molecules of BZD absorb in ultraviolet (UV)/visible rays that can be assayed by this property under well-standardized conditions to be able to apply the law of Beer–Lambert. Each molecule of BZD has an absorption maximum at which the measurement will be carried out. Prior extraction and calibration range are required for each assay. It is necessary that the molecule to be assayed has a characteristic spectrum and that there are not in the biological medium other molecules extracted under the same conditions and absorbing in the same ranges of wavelengths. The wavelength chosen for the BZD assay is generally between 210 and 350 nm. The ease of identification of a substance by its ultraviolet or visible spectrum depends on not only the number of bands present in the spectrum but also the width of these bands (a narrow band usually has a clearer maximum) or the possibility for a band to appear as a shoulder on a band more intense. Some authors have demonstrated that benzodiazepines extracted from samples can be detected using this optimized technique; for example, Doctor and McCord in 2013 [20] have demonstrated that this method overcomes the limitations of some immunoassay tests by providing lower LOD ranging (from 0.5 ng/mL to 127 ng/mL) and giving reproducible results over a wide range of concentrations of eleven different benzodiazepines and metabolites, except flunitrazepam and chlordiazepoxide where the LOD was below 50 ng/mL.

Generally, the use of this technique for BZDs characterization is much less appreciated, compared to other techniques; only some laboratories use this technique.

#### 4.2.5. Nuclear Magnetic Resonance Spectroscopy (NMR)

Nuclear magnetic resonance (NMR) spectroscopy is one of the most powerful techniques and versatile tools for retrieving detailed information about the structure, dynamics, and interactions of both organic and inorganic drugs. This technique has also proved to be useful in the qualitative and quantitative determination of the absorbing species such as BZD. Indeed, a study reported that Metizolam was detectable by NMR in hydrolyzed urine during the 46 hours period, with concentrations always lower than 11 ng/mL [42]. The resulting NMR spectra have very high information content, enabling the rapid detection and identification of analytes present in the sample such as urine [42]. Another favorable feature of NMR spectroscopy is that it is nondestructive, permitting the subsequent reanalysis of the sample by other methods [68].

There are different NMR spectroscopy experiments according to the studied nucleus and the type of information sought. We find NMR spectroscopy of ^1^H, ^2^H, ^13^C, ^15^N, ^31^P (the common nuclei studied using NMR in biomedical research), ^19^F, ^17^O, etc. Likewise, the advent of novel technologies for NMR spectrometers allows henceforth the routine acquisition of sophisticated one-dimensional (1D) and two-dimensional (2D) NMR spectra in relatively short periods of time on complex molecules [68].

The NMR experiments must be carried out under the same physicochemical conditions, since the various parameters are variable according to the temperature and the pH of the solutions. Each NMR signal is characterized by several magnitudes that are characteristic of the considered nucleus. These parameters give information about the number of nuclei, their environment, and their connectivity [68].

#### 4.2.6. Polarography

Polarography is a steady-state indicator method using intensity-potential curves plotted on a drop electrode of mercury. The solution transport of electroactive mercury species is due to diffusion. Following are the conditions of polarography:

- Half-wave potentials are the characteristic of the electroactive substance, hence the possibility of qualitative analysis
- The height of the bearings is proportional to the concentrations of these substances, hence the possibility of quantitative analysis

The conventional polarography has the main disadvantage of generating large capacitive currents due to the application of a potential to the electrode throughout the growth of the drop. This problem can be overcome by reducing the duration of application of the electrode potential used for current measurement, hence the idea of imposing short-term potential pulses [49].

There are two main versions of impulsion polarography: normal (PIN) and differential (PID). In PIN, from a constant value of potential, chosen in such a way that no electrochemical reaction takes place, pulses of variable amplitude are superimposed and incremented regularly from 1 to 2 mV so as to scan the potential area of interest. In PID, the amplitude of the pulse remains constant and it is the continuous potential that is incremented at each drop time to explore the window of potential interesting [49].

The main examples of applications include the following [50]:

- Trace analysis in the environmental field (heavy metals and organic pollutants)
- Analysis of bath components in the surface treatment industry (metals, additives, reducing agents, and impurities)
- Analysis of drugs or active ingredients in the pharmaceutical industry (vitamins, steroids, antibiotics, psychotropic drugs (1, 4 BZDs)) especially in the formulated products and also in the blood, serum, urine, and plasma.
- Analysis and quality control of food matrices such as ascorbic acid and vitamins and also contaminating compounds such as antibiotics, certain pesticides (Parathion), toxins (aflatoxin), or metals.
- Analysis and control in the plastics and polymers industry including additives used.

#### 4.2.7. Voltammetry

Voltammetry refers to the study of the intensity-potential curves of an electrochemical system. In this technique, a variable potential difference is applied across two electrodes of a measuring cell and the current flowing through the circuit is recorded. The cell contains a solution with chemical species that can give an oxidation or reduction reaction. Recent advances in electronics are at the basis of the development of this method, with the appearance of high-performance, highly sensitive equipment at affordable prices. Alone or coupled with other analytical techniques such as HPLC, GC, and TLC, it is used for the determination, qualitative or quantitative, of many organic substances like BZD [51, 69–71].

The variation of the potential imposed on the working electrode, as a function of time, can be linear continuous, differential with pulse, or in the form of square signal. The curves obtained, called voltammograms, are at the base of all the other electrochemical methods: amperometry, potentiometry, and coulometry.

According to Naggar et al. in 2012 [72], the use of sonogel-carbon electrode modified with 5% bentonite in comparison with other electrodes used in voltammetric determination of 1,4-benzodiazepines and with chromatographic and spectrophotometric methods, it can be considered as a time-saving procedure, low cost, selective, and sensitive, because the quantification and detection limits were calculated as 6.0 and 19.5 ng/mL for diazepam and chlordiazepoxide hydrochloride, respectively. And, according to Panahi et al. in 2018 [73], to improve the selectivity of the electrochemical techniques, the modification of the working electrode with selective adsorbate materials to selectively uptake the target species on the electrode surface is a very good suggestion.

#### 4.2.8. Potentiometry

Potentiometry is a measurement technique that passively evaluates the potential of a solution between two electrodes while affecting the solution in a minimal way. One of the electrodes is called the reference electrode (its potential remains constant), while the potential of the second (the working electrode) changes depending on the composition of the sample. The potential difference between the two electrodes then makes it possible to evaluate the composition of the sample with BZD [74–77].

The potentiometric technique generally involves working electrodes made selective for an ion of interest, so that the potential depends only on the activity of this ion of interest. The most widely used potentiometric electrode is the glass membrane electrode used in pH meters.

A variant of potentiometry is chronopotentiometry. This method consists in applying a constant current and measuring the potential as a function of time.

## 5. Conclusion

Various biological specimens and analysis methods have been conjointly used for the detection and quantification of psychotropic drugs, as described in this review. However, the most used biological samples are saliva, blood, and urine, whereas the commonly used analysis methods remain the quantitative and qualitative ones including HPLC and GC-MS. Toxicological screening using immunological tests, allowing the search for drugs such as BZDs, becomes more and more popular recently. The later tests will never replace those of the confirmation methods (HPLC, GC-MS, etc.), but they are complementary to establish a better and accurate diagnosis.

There is no ideal technique; hence, there is the need to choose several complementary methods according to the strategy adopted in each laboratory.

The choice of the toxicologist analyst should focus on separative methods, but it depends mainly on the vocation of his laboratory, priorities defined by the services local clinics, and technical and economic constraints in equipment and personnel. This situation requires a permanent dialogue between the doctor and the toxicologist analyst for optimal screening and follow-up.

## Figures and Tables

**Table 1:** Usual limits of detection (LOD) and quantification (LOQ) of benzodiazepines by gas chromatography (GC-MS).

Benzodiazepines	Method (GC-MS)				Sample type	Reference
	Instrument ID 9252		Instrument ID 9700			
	LOQ (ng/mL)	LOD (ng/mL)	LOQ (ng/mL)	LOD (ng/mL)		
Alpha-hydroxyalprazolam	5.53	5.53	5.70	5.70	Urine	[18]
Oxazepam	24.66	19.31	15.61	5.62		
Lorazepam	6.13	6.13	26.30	26.30		
Nordiazepam	7.72	7.72	14.98	14.98		
Temazepam	14.99	14.99	14.56	14.56		

Benzodiazepines^*∗*^	LOQ (ng/mL)		LOD (ng/mL)			
	0.52–58.47		1.58–177.2		Whole blood	[15]
Benzodiazepines^*∗∗*^	0.72–1.89		0.24–0.62			[13]

	DRUID^*∗∗∗*^ cutoff (ng/ml)					
Alprazolam, clonazeoam, flunitrazepam, lorazepam, nordiazepam	1				Oral fluid	[10]
Diazepam, oxazepam	5					

^*∗*^Diazepam, nordazepam, oxazepam, bromazepam, alprazolam, lorazepam, medazepam, flurazepam, fludiazepam, tetrazepam, chlordiazepoxide, clobazam, midazolam, flunitrazepam, 7-aminoflunitrazepam, triazolam, prazepam, nimetazepam, temazepam, lormetazepam, clonazepam, and camazepam. ^*∗∗*^7-Aminoclonazepam, *α*–OH–alprazolam, *α*–OH–midazolam, alprazolam, bromazepam, clonazepam, diazepam, flunitrazepam, lorazepam, midazolam, nitrazepam, nordazepam, oxazepam, temazepam, and triazolam. ^*∗∗∗*^The European Union project Driving under the Influence of Drugs (DRUID).

**Table 2:** Usual limits of detection (LOD), quantification (LOQ), and cutoff of some benzodiazepines by HPLC.

Benzodiazepines	HPLC		Sample type	Reference
	Cutoff (ng/mL)			
7-Aminoclonazepam	1		Oral fluid	[11]
Clonazepam	2.5			

	LOD (ng/mL)	LOQ (ng/mL)		
Nimetazepam	0.25	5	Urine	[17]
7-Aminonimetazepam	1	5		
Nitrazepam	0.125	1		
Flunitrazepam	0.125	0.25		
7-Aminoflunitrazepam	1	2.5		

Bromazepam, alprazolam, lorazepam, diazepam	0.01 (ng/*μ*L)	0.03 (ng/*μ*L)	Blood	[16]
